# Is Antiretroviral Therapy Cost-Effective in South Africa?

**DOI:** 10.1371/journal.pmed.0030060

**Published:** 2006-01-31

**Authors:** R. Scott Braithwaite, Joel Tsevat

## Abstract

Braithwaite and Tsevat discuss a new study that suggests that antiretroviral treatment is indeed cost-effective.

No resource allocation decision occurs in a vacuum. The decision to fund any public health intervention implies that those funds will be unavailable for alternative uses that may confer greater or lesser benefits. Even when funds are earmarked for a specific purpose, such as providing antiretroviral therapy (ART), questions persist: should more money be spent on pharmaceuticals and less on laboratory tests that monitor treatment response? should treatments be targeted toward individuals with a wide spectrum of disease stages, or should treatments be targeted exclusively toward individuals with late-stage disease?

The purpose of cost-effectiveness analysis is to enable the health and economic value from a particular policy decision to be compared with the value from alternative decisions. While many factors other than health and economic value need to be considered in the formulation of health policy (for example, ensuring equality in access to health services), such value is an important consideration. The cost-effectiveness of highly active antiretroviral therapy (HAART) has been studied widely in resource-rich countries, but data from resource-poor environments have been scarce [ 1] or are out of date [ 2, 3]. A new study by Motasim Badri and colleagues in *PLoS Medicine* on the cost-effectiveness of HAART in South Africa, therefore, constitutes an important addition to the literature [ 4].

## What is the Difference between Cost-Saving and Cost-Effective?

When an intervention is judged cost-effective, the implication is that its extra benefits justify its extra costs. If an intervention is cost-effective, this does not imply that the intervention is inexpensive (as the term “cost-effective” is often used in the vernacular) or cost-saving, since many cost-effective interventions are expensive. When an intervention is judged cost-saving, the intervention actually saves money.

## What Did the Authors Do and Find?

The authors compared the costs and benefits between individuals receiving HAART and individuals not receiving HAART in a cohort of individuals infected with HIV in South Africa. Patients who participated in clinical trials and who received at least three ART drugs were considered the “treated arm” of the study. Patients who did not participate in these trials and who never had access to ART throughout the study period, but who received other HIV-related care, constituted the sample from which a “comparator” group was identified.

No resource allocation decision occurs in a vacuum.

Because the authors did not conduct a randomized trial (they simply compared two cohorts of patients), they attempted to statistically adjust for important differences between the cohorts, such as age, socioeconomic status, and CD4 count. The authors concluded that individuals on HAART not only live longer but may have lower costs, depending on what price structure is assumed for HAART. With the current pricing of HAART (US$730 per patient-year), the incremental cost-effectiveness of HAART versus no HAART was US$1,622 per additional life-year gained for individuals without AIDS. For individuals with AIDS, HAART would be cost-saving at that price. With the lower prices for HAART that would likely result from local manufacturing (US$181 per patient-year), the incremental cost-effectiveness of HAART would be even more favorable at US$675 per life-year gained for individuals without AIDS, and HAART would remain cost-saving for individuals with AIDS.

The primary result of this analysis, an estimation of HAART's additional cost per life-year gained, was judged for its economic attractiveness by comparing it with a guideline that considers the probable size of a country's health budget (each disability-adjusted life-year should cost no more than two times the yearly gross domestic product). But this guideline is not an intrinsic tenet of cost-effectiveness analysis; rather, it resembles the “rule of thumb” suggested by the World Health Organization for interpreting incremental cost-effectiveness ratios (see chapter 5 of [ 5]). While this decision rule reflects current disparities in health budgets, it may be criticized because it codifies the notion that life in wealthier countries is worth more than life in poorer countries.

## Strengths and Weaknesses of the Study

The primary strength of Badri and colleagues' study is its incorporation of prospectively measured cost and outcome data from the target population. An additional strength is its analysis of multiple-drug cost scenarios. Some cost-effectiveness studies presume a fixed or narrow range of costs, which is inappropriate because prices may fluctuate dramatically in an unanticipated fashion, with commensurately large fluctuations in cost-effectiveness (e.g., the 20-fold decrease in the price of HAART that has been observed over the past five years).

Limitations of the study include the lack of randomization, the questionable methods for adjusting for differences in patient characteristics, and the atypical methods for calculating life expectancies and costs. In particular, adjusting for CD4 count may have biased the analysis against HAART because individuals on HAART often experience rapid elevations in CD4 count. Therefore, this analysis may have compared individuals off therapy with individuals on therapy who initially had lower CD4 counts, and were far sicker. Estimates of the life expectancies for patients taking or not taking HAART and for patients with AIDS versus no AIDS were based on median disease progression times, which were themselves estimated, rather than on areas under the survival curves.

Another limitation of the study was its brief time frame for considering costs. While individuals treated with HAART will initially have lower expenditures because of fewer hospitalizations, over their lifetimes their expenditures would be expected to increase as HAART becomes less effective due to resistance. These assumptions may have biased the analysis in favor of HAART. Finally, the authors' decision to avoid discounting results is debatable, and may complicate comparing their results with those from other studies [ 6]. Discounting reflects the notion that money or benefits that arrive today may be valued more than similar money or benefits that arrive in the future.

## Can Cost-Effectiveness Analysis Be Used to Deny Treatment?

Some authors have invoked cost-effectiveness analysis to question whether HAART should be provided in resource-poor areas [ 7–9]. However, it is important to note that cost-effectiveness analysis by itself never implies that particular programs should or should not be funded—it only allows their relative benefits to be ranked. If health budgets are insufficient, many health interventions that deliver great benefits will appear unfavorable because the budget will have been exhausted by competing uses that offer even greater value. Therefore, when an obviously beneficial and life-saving service is deemed insufficiently cost-effective, it is an indictment of the parsimony of the health budget itself, not of the method of cost-effectiveness analysis.

## Policy Implications

The analysis by Badri and colleagues suggests that providing HAART in South Africa is likely to be relatively cost-effective for individuals without AIDS and even cost-saving for individuals with AIDS, assuming current drug prices and the “rule of thumb” for interpreting cost-effectiveness ratios suggested by the World Health Organization. Moreover, if the price of HAART medications decreases further through local production, the cost-effectiveness of HAART for individuals without AIDS would become substantially more favorable. Therefore, these results support providing HAART to individuals with HIV in South Africa.

## Abbreviations

ART: antiretroviral therapy
HAART: highly active antiretroviral therapy
